# Serratia marcescens-Induced Pericarditis Leading to Rapid-Onset Cardiac Tamponade in a Patient With Underlying Malignancy

**DOI:** 10.7759/cureus.98656

**Published:** 2025-12-07

**Authors:** Ashirbad Acharya, Anurag Karki, Narayan Bhurtel, Simran Pradhan, Puja Thapa, Yogesh Pandey, Suraj Shrestha, Ricardo Conti

**Affiliations:** 1 Internal Medicine, AdventHealth Orlando, Orlando, USA; 2 Internal Medicine, Kathamndu University School of Medical Scineces, Kathmandu, NPL; 3 Department of Research and Development, National Open College, Lalitpur, NPL; 4 Health Administration, University of Central Florida, Florida, USA; 5 Internal Medicine, Tribhuvan University, Institute of Medicine, Maharajgunj Medical Campus, Kathmandu, NPL

**Keywords:** cardiac tamponade, iv drug-use, serratia, serratia marcescens pericarditis, small-cell lung carcinoma

## Abstract

*Serratia marcescens* is a Gram-negative bacterium that mostly causes urinary tract infections, respiratory infections, and catheter-related bloodstream infections. It rarely causes pericarditis, especially leading to tamponade, which is exceptionally rare. We describe a case that had a history of intravenous drug usage and presented with dyspnea and chest pain. Small cell lung carcinoma and a newly developed moderate pericardial effusion with echocardiographic indications of imminent tamponade were discovered during imaging. Pericardial fluid cultures identified Serratia marcescens. The patient was treated successfully with pericardial fluid drainage followed by antibiotics. This case emphasizes the importance of considering unusual organisms like *S. marcescens* when treating pericardial infections, particularly in patients who are at high risk. Early identification and focused treatment are crucial for the management of complications like cardiac tamponade.

## Introduction

*Serratia marcescens* is a Gram-negative, facultative anaerobic bacterium that is an opportunistic pathogen causing various healthcare-associated infections [1], including urinary, respiratory, and catheter-related infections, especially in immunocompromised and intravenous drug users [2,3]. Cardiac involvement is rare; when it occurs, it most commonly presents as infective endocarditis, accounting for a small fraction of all endocarditis cases (e.g., Serratia caused 14% of all addict-associated endocarditis) [4]. Serratia pericarditis is exceptionally rare, with one reported case in 1983. [5]. We report a rare case of pericarditis caused by Serratia marcescens, leading to cardiac tamponade. This article was previously presented as a poster at the 2025 Society of Hospital Medicine Converge Meeting on April 24, 2025.

## Case presentation

**Figure 1:**
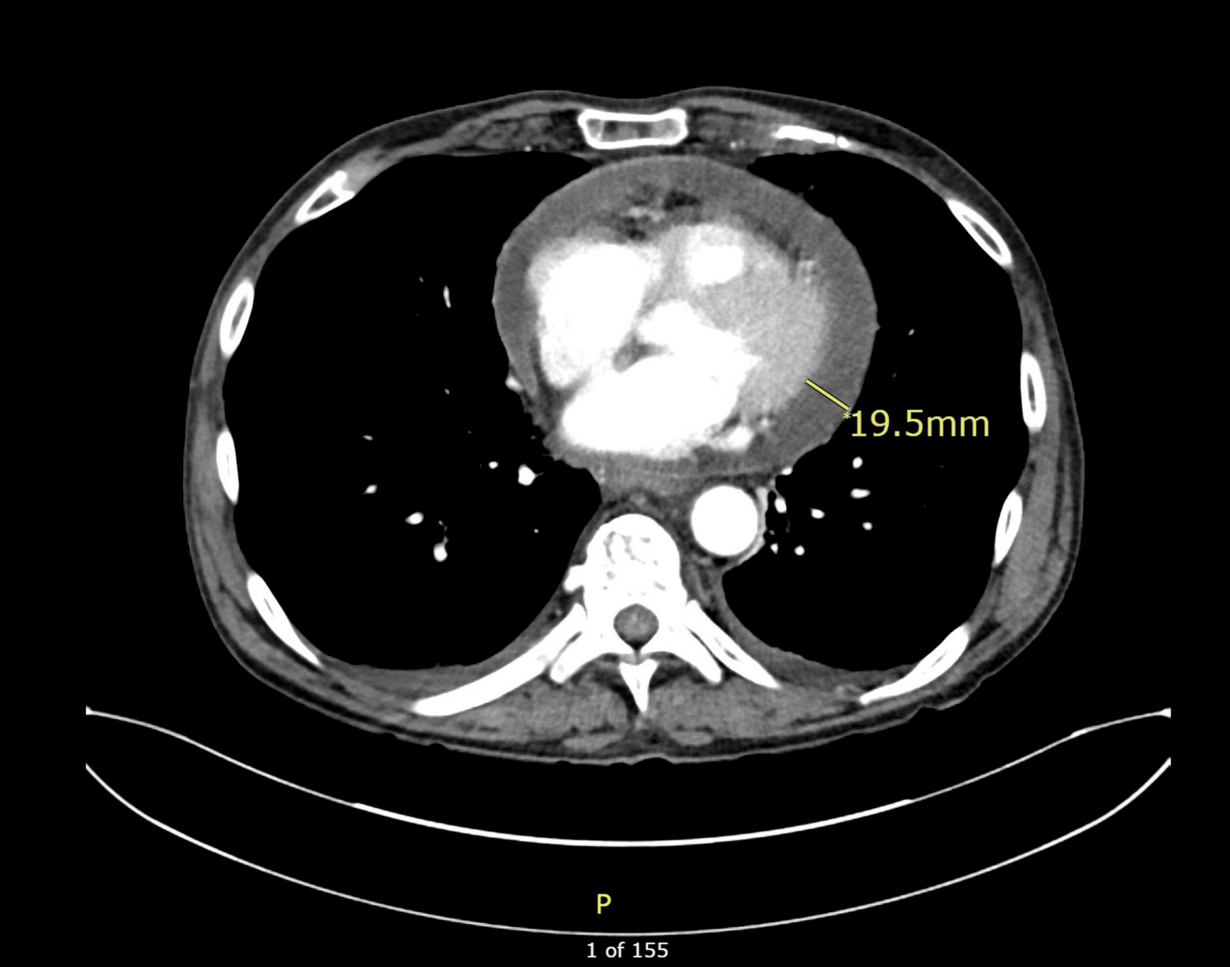
Contrast-enhanced axial CT scan demonstrating pericardial effusion The measurement marker indicates pericardial fluid thickness of approximately 19.5 mm.

**Figure 2:**
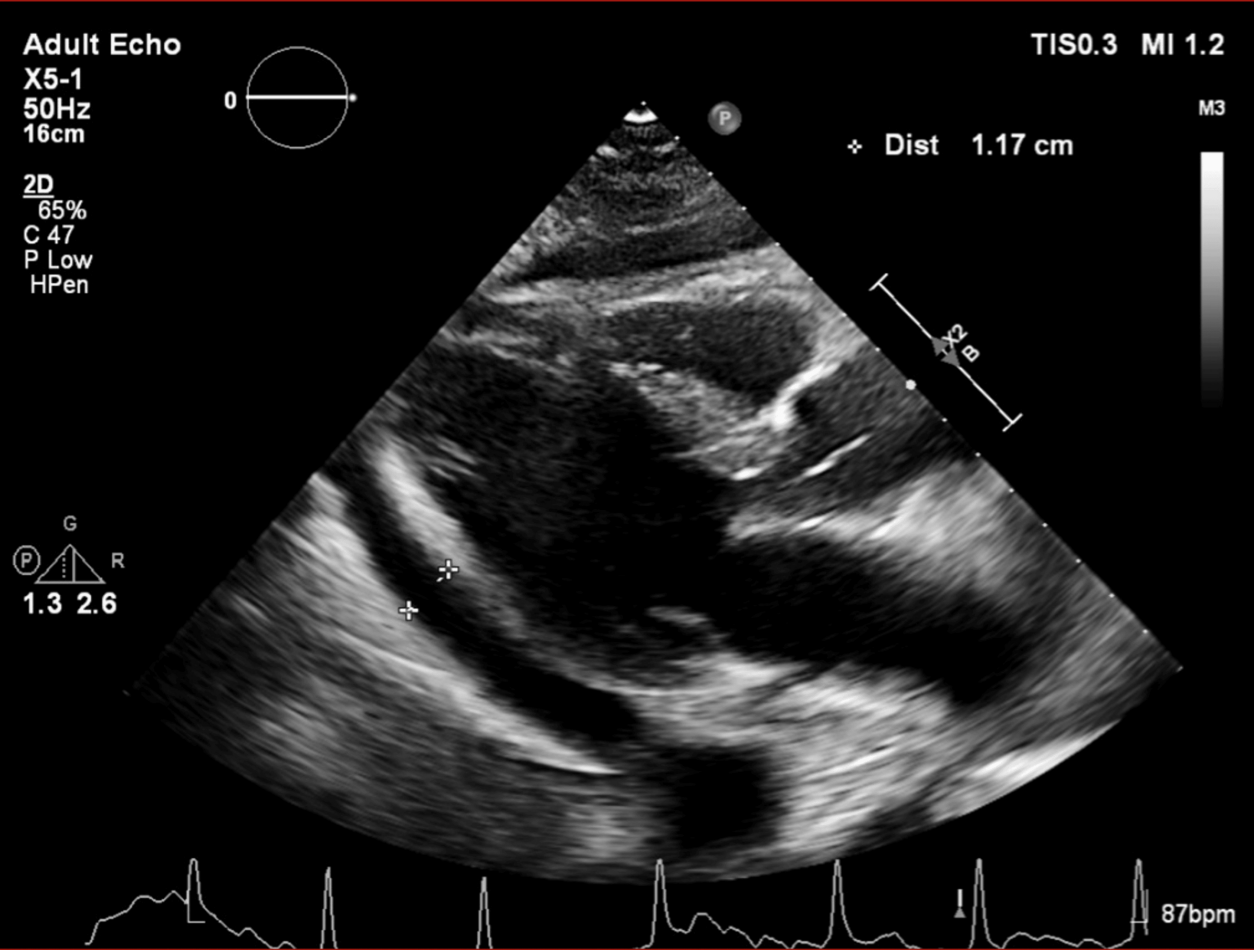
Transthoracic echocardiogram (parasternal long-axis view) showing pericardial effusion The measurement markers demonstrate a pericardial fluid collection of approximately 1.17 cm.

**Figure 3:**
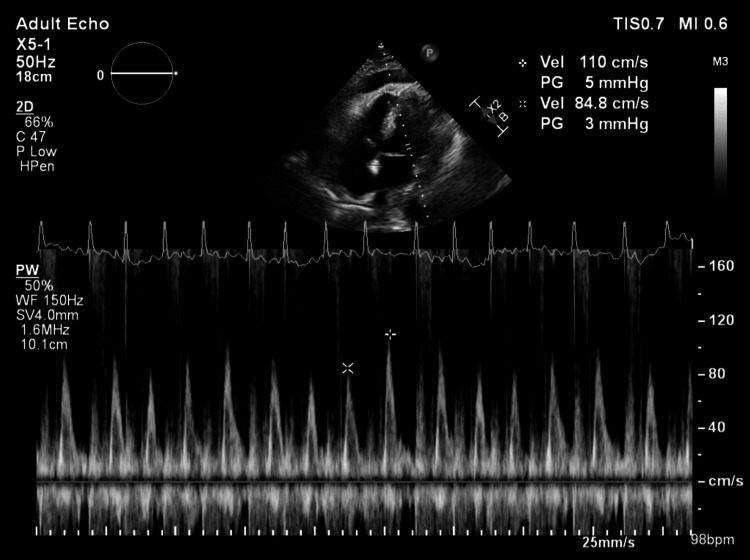
Transthoracic echocardiography with pulsed-wave Doppler across mitral inflow in apical four-chamber view demonstrating increased mitral transvalvular velocity variation during respiration, suggestive of cardiac tamponade The marked arrows show peak velocity of 110 cm/s (5 mmHg gradient) during inspiration and 84.8 cm/s (3 mmHg gradient) during expiration, showing increased respiratory variation in mitral inflow velocities, which is suggestive of tamponade physiology.

A 66-year-old male with a history of intravenous (IV) drug use presented with a day history of acute onset chest pain and shortness of breath. The physical exam was unremarkable. Toxicology screen identified cocaine, cannabinoids, and fentanyl. X-ray showed a hilar mass, which on CT showed a right upper lobe nodule concerning for malignancy and mediastinal and hilar adenopathy. EKG showed diffuse non-specific ST-elevations. A urine culture revealed significant colonies of Klebsiella pneumoniae. The patient was started on trimethoprim-sulfamethoxazole. Bronchoscopic biopsy was subsequently performed, which showed small cell lung cancer and Actinomyces in lung culture. Initially absent on the first CT, follow-up CT 2 days later for staging revealed a previously undetected moderate pericardial effusion, as shown in Figure 1. Although the exam demonstrated no paradoxical pulse or tachycardia, the echocardiography showed pericardial effusion, as shown in Figure 2, and mitral transvalvular velocity variation during respiration, suggesting cardiac tamponade, as shown in Figure 3. However, it did not show any evidence of vegetations. The patient had a pericardial window done, and 300 ml of pericardial fluid was initially removed. The pericardial fluid culture was positive for Serratia marcescens, which was subsequently managed with piperacillin-tazobactam 4.5 g 8 hourly. However, blood cultures, which were negative on admission, remained so on re-culture. Pericardial biopsy did not reveal any malignancy. He started chemotherapy and was later discharged on piperacillin-tazobactam to complete 6 weeks of therapy.

## Discussion

*S. marcescens* is a facultative Gram-negative bacillus that causes a wide spectrum of infections, including those involving the urinary tract, respiratory system, biliary tree, and catheter-associated sites [1]. However, its involvement in pericardial disease is exceptionally rare, especially in the absence of bacteremia or endocarditis. Bacterial pericarditis itself is uncommon, with *Streptococcus pneumoniae* and *Staphylococcus aureus* being the most frequent organisms [6,7]. Endocarditis is often observed when the heart is involved, but isolated pericarditis, particularly due to *S. marcescens*, is exceedingly rare. Our literature review revealed only one reported case of pericarditis due to S. marcescens, with the patient having disseminated infection and bacteremia [5]. Isolated pericarditis due to *S. marcescens* was not found during our review. This highlights the need to consider rare pathogens like *S. marcescens* in pericardial effusion, especially in patients with known risk factors.

Acute pericarditis in the developed world is usually idiopathic or of viral origin, and large effusions are generally linked to malignancy, tuberculosis, hypothyroidism, or are idiopathic [8]. In our case, although the patient had a history of lung cancer, both pericardial biopsy and cytology were negative for malignant involvement. Furthermore, there were no signs of common precipitating causes, such as blunt trauma, aortic dissection, or cardiac rupture. Given this, an infectious etiology was suspected.

Notably, the patient had a history of intravenous drug use (IVDU), a recognized risk factor for Serratia infections [3]. While IVDU typically predisposes individuals to bacteremia or endocarditis, our case reinforces that localized infections such as pericarditis can occur independently.

*S. marcescens* is known for its complex antibiotic resistance mechanisms. It often expresses inducible AmpC and extended-spectrum beta-lactamase (ESBLs), making it resistant to several classes of antibiotics, including penicillins and cephalosporins [1,9]. While carbapenems have traditionally been the first-line agents for Serratia infections, rising resistance to carbapenems is concerning [10]. Recent studies suggest that alternatives such as piperacillin-tazobactam or fourth-generation cephalosporins can be used effectively, especially in non-ESBL-producing strains. In our case, piperacillin-tazobactam was used successfully. This aligns with recent findings advocating for carbapenem-sparing approaches where susceptibility permits. [11] Nevertheless, the challenge in treating Serratia infections lies not only in drug resistance but also in the organism’s ability to evade host defenses through biofilm formation and latent persistence [1].

There have been reported instances of *S. marcescens* recurrence despite apparent resolution, even after prolonged periods. One patient experienced recurrent implantable cardioverter-defibrillator (ICD) infections, while another developed pericardial effusion post-cardiac transplant and later presented with sternal osteitis caused by the same organism 15 years later [12,13]. These cases underscore Serratia’s potential for long-term latency and recurrence, highlighting the need for prolonged monitoring even after apparent clinical recovery.

Given its aggressive nature, rapid progression, and high mortality, early diagnosis and timely initiation of targeted antibiotics are paramount in bacterial pericarditis [5,7]. Clinicians should maintain a high index of suspicion in at-risk populations who present with large or rapidly accumulating pericardial effusions.

## Conclusions

This case illustrates the possibility of Serratia pericarditis as a cause of rapidly growing pericardial effusion. Risk factors such as IV drug use should raise suspicion toward the diagnosis of infectious etiology. Early diagnosis and treatment are crucial to prevent complications such as cardiac tamponade.
